# Immune Checkpoint Inhibitors versus VEGF Targeted Therapy as Second Line Regimen in Advanced Hepatocellular Carcinoma (HCC): A Retrospective Study

**DOI:** 10.3390/jcm9092682

**Published:** 2020-08-19

**Authors:** Anwaar Saeed, Hannah Hildebrand, Robin Park, Mohammed Al-Jumayli, Saqib Abbasi, Tina Melancon, Azhar Saeed, Raed Al-Rajabi, Anup Kasi, Joaquina Baranda, Stephen Williamson, Weijing Sun

**Affiliations:** 1 Department of Medicine, Division of Medical Oncology, Gastrointestinal Oncology Program, Kansas University Cancer Center, Kansas City, KS 66205, USA; hhildebrand@kumc.edu (H.H.); sabbasi@kumc.edu (S.A.); tmelancon@kumc.edu (T.M.); ral-rajabi@kumc.edu (R.A.-R.); akasi@kumc.edu (A.K.); jbaranda@kumc.edu (J.B.); swilliam@kumc.edu (S.W.); wsun2@kumc.edu (W.S.); 2 MetroWest Medical Center, Tufts University School of Medicine, Framingham, MA 02111, USA; robin.park@mwmc.com; 3 Department of Medicine, Division of Medical Oncology, University of South Florida, Moffitt Cancer Center, Tampa, FL 33620, USA; mjumayli@gmail.com; 4 Department of Pathology and Laboratory Medicine, Kansas University Medical Center, Kansas City, KS 66160, USA; asaeed2@kumc.edu

**Keywords:** hepatocellular carcinoma, immunotherapy, checkpoint inhibitors, Program Death Ligand 1, targeted therapy, multi-tyrosine kinase inhibitors

## Abstract

Several targeted agents including multi-tyrosine kinase inhibitors (mTKIs) and immunotherapy (IO) agents have been approved for use beyond the frontline setting in patients with advanced hepatocellular carcinoma (HCC). Due to lack of prospective head-to-head comparative trials, there is no standardized way for alternating those agents beyond frontline. Therefore, we performed a retrospective review of the Kansas University (KU) cancer registry to determine whether IO may be superior to non-IO therapy. Patients with advanced HCC were divided into two groups based on the second-line systemic regimen received (IO vs. non-IO). Progression-free survival (PFS) and overall survival (OS) were calculated under the Kaplan–Meier and Cox proportional hazards models. No statistically significant differences in PFS and OS were found, although a non-significant delayed separation in the survival curve favoring IO was identified (median PFS 3.9 months vs. 3 months; median OS 10 months vs. 10 months respectively for IO vs. non-IO). This retrospective analysis is one of the earliest and largest studies comparing second-line IO and non-IO therapies thus far reported. Future studies should aim to define specific biomarkers for response prediction and treatment optimization based on individual patient and tumor characteristics. Furthermore, combinatorial therapeutic strategies is an evolving approach showing early promising signal.

## 1. Introduction

Hepatocellular carcinoma (HCC) is one of the most common types of cancer worldwide and is the third leading cause of cancer-related death [1,2]. HCC is a primary tumor of the liver that commonly develops in the setting of chronic liver disease, particularly in patients with cirrhosis and chronic hepatitis B virus or hepatitis C virus infection [3]. Although the incidence of HCC is decreasing in some areas due to the increase in availability of the hepatitis B vaccine, HCC accounts for an estimated 600,000 deaths globally per year, and mortality rates continue to rise. Because it is typically diagnosed late in its course, and curative treatment is often not feasible for greater than 80% of patients, the median survival following diagnosis is only approximately 6 to 20 months [1,2]. Therefore, patient selection and treatment approaches should be adequately defined in order to prolong survival in this fatal disease.

Although the mainstay of therapy for HCC is surgical resection, the majority of patients are ineligible because of tumor extent or underlying liver dysfunction [4]. Until 2008, no effective systemic therapy had existed for patients with advanced-stage HCC or for those failing local therapies; however, there has been a resurgence of interest and enthusiasm for systemic therapy with the emergence of data showing that molecular-targeted agents like sorafenib improve survival compared to best supportive care alone. Lenvatinib is non-inferior to sorafenib in the first-line setting based on the REFLECT trial [5]. Furthermore, most recently based on the IMBrave150 trial in which atezolizumab plus bevacizumab demonstrated superior survival compared to sorafenib (hazard ratio (HR), overall survival (OS), 0.58, 95% CI, 0.42–0.79; progression-free survival (PFS), 0.59, 95% CI, 0.47–0.76), this combination regimen was approved for use in the frontline setting [6]. 

Subsequently, clinical trials for a number of different agents demonstrating survival benefit over placebo were published in the second-line setting. In the RESORCE and CELESTIAL trials respectively, regorafenib and cabozantinib demonstrated OS benefit compared to placebo (HR, 0.63, 95% CI, 0.50–0.79; HR, 0.76, 95% CI, 0.63–0.92) [7,8]. In the REACH-I trial, ramucirumab initially failed to demonstrate a survival benefit in the unselected population, but subgroup analysis suggested prolonged survival in the alpha-fetoprotein (AFP)-high group, prompting a subsequent trial, REACH-II, which confirmed superior OS in the AFP-high population (HR, 0.71, 95% CI, 0.53–0.94) [9,10]. A number of immune checkpoint inhibitors (IOs) have been studied in HCC including pembrolizumab, nivolumab, and atezolizumab with modest success. The KEYNOTE-240 trial demonstrated pembrolizumab’s OS benefit over placebo and became the basis of its approval (HR, 0.781, 95% CI, 0.61–0.99); furthermore, the CheckMate-040 trial demonstrated durable response and led to the approval of niovlumab with or without ipilimumab [11,12,13]. Second-line therapy is an option for patients whose tumors progress while on first-line therapy and whose performance status and liver function are sufficient to tolerate it. The optimal approach to second-line therapy has not been established. For patients who are ineligible for or without access to clinical trials, options include multi-targeted tyrosine kinase inhibitors (i.e., regorafenib and cabozantinib if they were not given as a first-line therapy), the immune checkpoint inhibitors nivolumab and pembrolizumab, as well as the anti-angiogenic monoclonal antibody ramucirumab with the latter reserved for patients with elevated AFP > 400 per the REACH-II data [14].

A superior regimen among the second-line options has not been established, and no biomarkers exist to guide treatment selection of one agent over another. The available data suggest a modest degree of antitumor efficacy for several conventional cytotoxic agents and/or combination drug regimens. However, the appropriate selection of patients for cytotoxic chemotherapy, especially in view of the advances in molecularly targeted therapies and immunotherapy, is not clear [15]. The purpose of this study is to compare the efficacy of the available second-line therapies—specifically immune checkpoint inhibitors (IO), defined as the use of any programmed cell death protein 1 (PD-1) inhibitors nivolumab or pembrolizumab—compared to vascular endothelial growth factor receptor (VEGFR2) multi-tyrosine kinase inhibitors (mTKIs) or monoclonal antibodies (non-IO), namely sorafenib, regorafenib, cabozantinib, and ramucirumab, for the treatment of advanced HCC and to investigate for potential patient subgroup variables that influence efficacy.

## 2. Experimental Section

### 2.1. Study Design

We retrospectively reviewed medical charts from the University of Kansas Cancer Center registry following approval from the Institutional Review Board. Our data included 98 patients with an established diagnosis of HCC who had disease progression on any first-line systemic therapy. Patients were divided into two groups depending on their second-line regimen (IO vs. non-IO). Primary outcomes included progression-free survival (PFS) and overall survival (OS). PFS was defined as the time from initiation of second-line therapy to failure of treatment, defined as progression of disease or death, whichever came first. OS was defined as the time from initiation of second-line therapy to time of death. Disease progression was determined based on RECIST version 1.1. Secondary analyses included the evaluation of PFS and OS in the hepatitis C infected subgroup.

### 2.2. Subjects and Procedures

Patients with HCC with age greater than 18 were eligible if they were diagnosed with unresectable HCC from 2010 to 2018 and had disease progression on first-line systemic therapy. Patients were included only if their second-line treatment was either an IO (as defined above) or an mTKI or anti-VEGFR2 (as defined above). Data including age, gender, Eastern Cooperative Oncology Group (ECOG) performance status, Child–Pugh score, Model for End-Stage Liver Disease (MELD) score, and baseline AFP at the start of second-line treatment were extracted; the underlying etiology of liver disease was identified when data were available; data were obtained regarding whether the preceding first-line therapy was sorafenib, lenvatinib, nivolumab, or other IO agents. Data were also obtained as to whether surgery or liver-directed local-regional therapies preceded systemic therapy, and whether patients had extra-hepatic metastatic disease at progression.

### 2.3. Statistical Data Analysis

Baseline characteristics were compared between those who received IO versus non-IO for second-line therapy using pooled *t*-tests for factors including age, MELD score, and AFP. ANOVA was used to compare etiologies of liver disease, and chi-square analysis was used to compare gender, ECOG performance status, Child–Pugh score, previous systemic therapy and preceding surgery or local therapies, and metastatic disease at progression.

PFS and OS were calculated using the Kaplan–Meier and corresponding Cox proportional hazard models. Survival curves were compared using the Log-rank test. Median PFS and OS and corresponding 95% confidence intervals (95% CI) were determined from Kaplan–Meier product limit estimates from the associated survival curves. Hazard ratios (HRs) were determined using the Cox proportional hazard regression analysis.

## 3. Results

A total of 98 patients were included in the analysis. First- and second-line treatment modalities are summarized in Table 1. The majority of patients received sorafenib and lenvatinib in the first- line setting (89.8%), and a subset of patients received nivolumab and subsequently received sorafenib (10.2%).

Mean age was 61.9, and 21.4% were female. No statistically significant difference existed among ECOG performance status, MELD score, and AFP at baseline between IO and non-IO patients. The predominant etiology of underlying liver disease was hepatitis C (56.1%) followed by alcohol (28.6%). No statistical difference existed for underlying liver disease etiology among the two groups. Most patients received frontline sorafenib in both groups, although a higher proportion of patients received frontline sorafenib in the non-IO subset (98.2% vs. 73.2%, *p*-value < 0.01). A higher proportion of patients in the IO subset initially received regional therapy (47.4% vs. 70.7%, *p* = 0.03) (Table 2). The baseline characteristics of the hepatitis C virus (HCV) positive HCC group are summarized in Table 3. 

**Figure 1:**
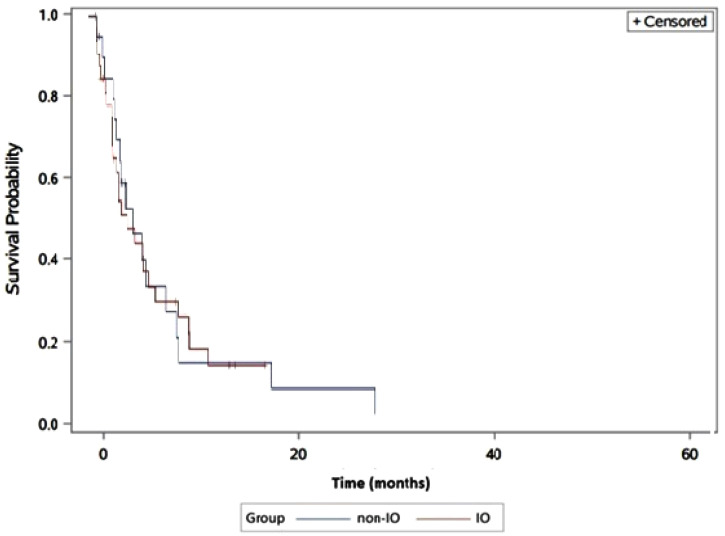
Progression-free survival in immunotherapy (IO) versus non-IO subgroups. The blue line represents the non-IO whereas the red line represents the IO population.

Patients in the IO population had a median PFS of 3.9 months (95% CI: 2.3–6.9) compared to 3.0 months (95% CI: 2.1–5.4) for the non-IO population. No statistically significant difference for PFS was found between the two groups (Log-rank test *p* = 0.07, Cox proportional HR 1.11 (95% CI: 0.90–1.31)) (Figure 1).

**Figure 2:**
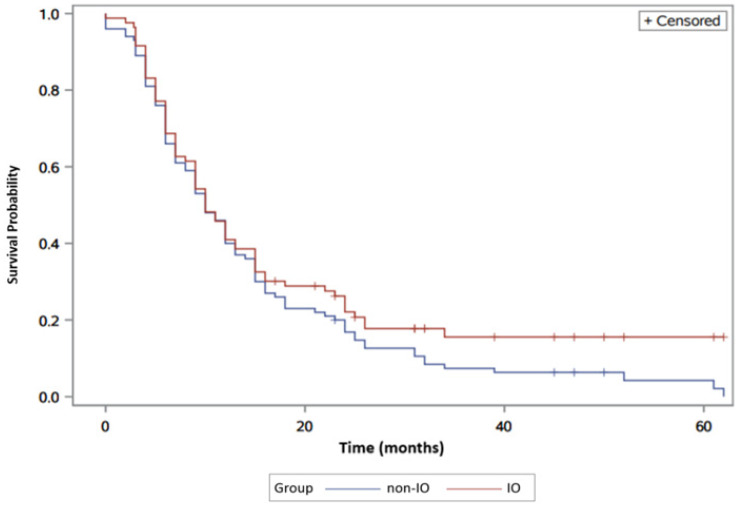
Overall survival in IO versus non-IO patients. The blue line represents the non-IO whereas the red line represents the IO population.

Moreover, the median OS between IO versus non-IO was 10 months (95% CI: 8–12) and 10 months (95% CI: 9–13) respectively. No significant difference was found for OS between the two populations (Log-rank *p* = 0.31, Cox proportional HR 0.98 (95% CI: 0.85–1.12)). It is important to note, however, the delayed separation of the two curves favoring the IO population (Figure 2).

**Figure 3:**
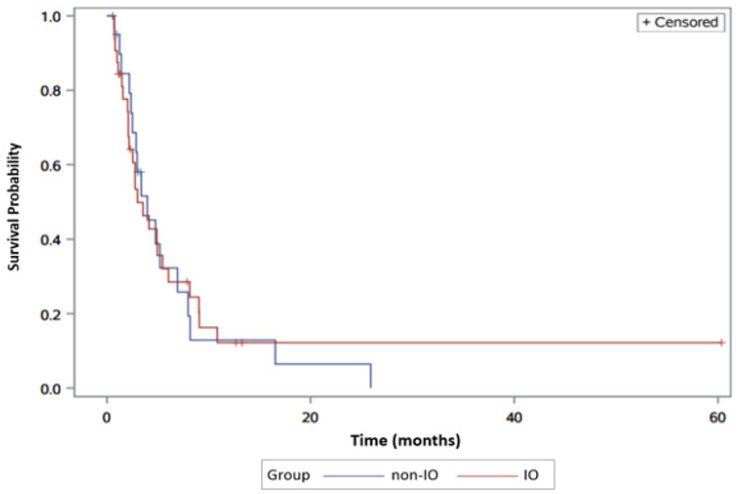
Progression-free survival in hepatitis C virus (HCV)-positive hepatocellular carcinoma (HCC) patients in IO versus non-IO subgroups. The blue line represents the non-IO whereas the red line represents the IO population.

Hepatitis C was the underlying etiology of liver disease in 56.1% of patients. Among these patients, 33 received second-line non-IO therapy and 22 received IO therapy. Median progression- free survival was 3.2 months (95% CI, 1.5–4.7), compared to 3.1 months (95% CI, 1.4–4.9). No statistically significant difference for PFS was found between the two groups (Log-rank test *p* = 0.90) (Figure 3).

**Figure 4:**
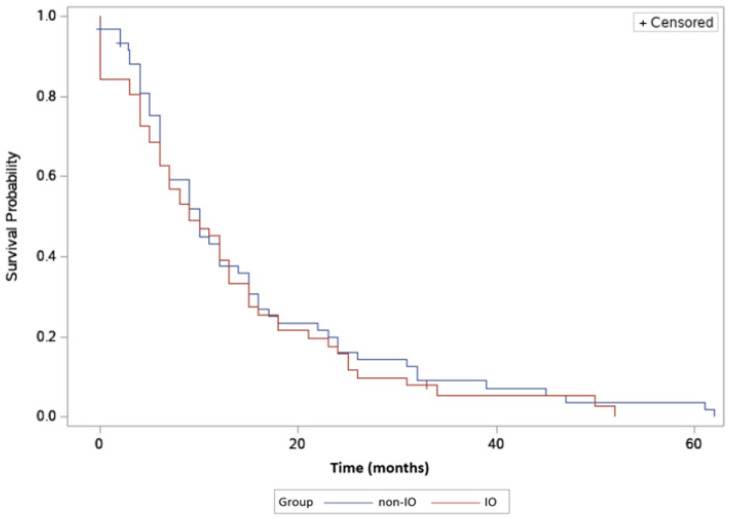
Overall survival in HCV-positive HCC patients in IO versus non-IO subgroups. The blue line represents the non-IO whereas the red line represents the IO population.

Overall survival was nine months (95% CI, 6–12) in the non-IO group, compared to 8.5 months (95% CI, 6–11) in the IO group. No statistical difference was identified in overall survival in this hepatitis C subgroup (Log-rank test *p* = 0.52) (Figure 4).

## 4. Discussion

The therapeutic armamentarium for advanced HCC has greatly expanded since the initial approval of sorafenib in 2008. In the first-line setting, the REFLECT trial demonstrated the non-inferiority of the relatively well-tolerated lenvatinib to sorafenib, leading to its approval for first-line therapy [5]. Most recently, and perhaps most importantly, atezolizumab plus bevacizumab was the first regimen to demonstrate improved survival over sorafenib in the first-line setting and has become the new standard of care [6]. Furthermore, in the past two years, multiple agents have been approved for second-line therapy, including mTKIs such as regorafenib, cabozantinib, and VEGFR2 inhibitor ramucirumab, as well as PD-1 inhibitors nivolumab and pembrolizumab that have shown increased clinical benefit in this setting [8,10,11,13,16]. Among these options, however, the optimal second-line treatment for unresectable HCC remains a subject of continuous debate. However, the sheer number of FDA-approved second-line therapeutic agents for advanced HCC makes direct head-to-head comparisons of all possible permutations challenging, perhaps impossible. Therefore, a retrospective analysis is a useful tool to evaluate the comparative efficacy, the results of which may inform subsequent prospective trials, which led us to conduct this study.

To our knowledge, this is one of the largest retrospective studies reported comparing second- line systemic therapeutic agents for unresectable HCC. Furthermore, our study is unique in that a subset of patients in the non-IO population received sorafenib as second-line therapy. Based on our analysis, no significant differences were found in the PFS and OS between the IO and non-IO populations, although it is worth noting that a delayed separation in OS favoring the IO subgroup was found.

Immunotherapy has demonstrated durable responses with prolonged OS, which can at times be striking per previous clinical trials. The durable responses of immunotherapy are manifested as splitting of the “long tails” of the OS curves. A subset of patients who have survived beyond three years, which constitutes 18% of patients, seem to have sustained survival to the end of study when compared to all patients eventually dying in the TKI subgroup. This is verified by a similar subset of patients who have sustained PFS represented by the inverse plateau of the PFS curve among IO patients. This delayed separation of the Kaplan–Meier survival curve is a unique response pattern that is consistent with other trials studying the checkpoint inhibitors subgroup of immunotherapy agents [17,18]. Unfortunately, our study was not powered to detect the difference statistically. Future trials may need to consider alternate endpoints of drug efficacy such as durable control rates. This observation may also bring the question of whether reversed sequence of drug delivery means IO followed by mTKIs might be more effective for those subset patients with sustained PFS from IO. Developing valid predictive biomarkers for this endpoint continues to be an important venue of research. To this end, putative predictive biomarkers including conventional tumor markers, immune checkpoint molecules, tumor mutational burden (TMB), and circulating tumor cell free DNA (cfDNA) are under investigation and require validation before being used in clinical settings.

Previous studies have supported a strong mechanistic rationale for combining IO with anti-VEGF/TKI in achieving greater clinical benefit. The basis of this clinical benefit may be attributable to the immunomodulatory effects of anti-VEGF/TKI, including enhanced dendritic cell maturation, increased effector T-cell and decreased regulatory T-cell, and myeloid-derived suppressor cell tumor infiltration, which lead to a tumor microenvironment favorable for generating anti-tumor immune responses, which in turn leads to potentiation of IO efficacy [19,20,21,22]. This hypothesis is supported by the success of the recent phase III trial evaluating the combination of atezolizumab and bevacizumab as first-line regimen compared to sorafenib. This combination demonstrated a statistically significant and clinically meaningful improvement in both OS and PFS in patients with unresectable HCC who did not receive prior systemic therapy. The reported median OS with the combination was not reached compared to 13.2 months with sorafenib. In addition, the combination was also more tolerable with better patient-reported outcomes and better quality of life as compared to sorafenib [6]. The favorable efficacy outcome of this combination is consistent with the results of other novel combinations like pembrolizumab (Keytruda) and lenvatinib evaluated in the phase 1b KEYNOTE-524. The above results have prompted the FDA to grant approval to the atezolizumab plus bevacizumab combination regimen in the first-line setting [23]. Triple therapy with IO doublets and mTKIs for advanced HCC has also been recently explored and yielded positive outcomes as with the CheckMate 040 trial. In this trial, patients were randomized to receive cabozantinib plus nivolumab with or without ipilimumab. Overall response rate was 17% with the double therapy and 26% with the triple therapy. Median PFS was 5.5 months for double therapy and 6.8 months for triple therapy. Although treatment-related adverse events were higher in the triple therapy, the majority of adverse events were manageable [24]. In addition, other trials, such as the ongoing CAMILLA phase I/II trial, are evaluating the combination of cabozantinib plus durvalumab in GI malignancies, including an HCC cohort [25]. Per the positive outcome of those recent trials, the novel combination of IO with VEGF-targeted therapy is changing the treatment landscape for patients with unresectable HCC and potentially the therapeutic options for other GI malignancies.

There is a well-known association between chronic HCV and immune exhaustion, as well as between immune checkpoint inhibitors and restoration of T-cell immunity in in vitro and in vivo studies [26,27,28,29]. Furthermore, the KEYNOTE-224 and CheckMate-040 trials demonstrated the safety and efficacy of PD-1 inhibitors in HCC without exacerbation of HCV flares [13,30]. However, whether checkpoint inhibitors or VEGF-targeted agents were superior in comparison remains in question, which prompted our analysis in the HCV-positive HCC subgroup. Nonetheless, our results show no significant difference in the HCV-positive HCC subgroup. 

There are several limitations of our study. First, the single-institution, retrospective nature of the study, as well as the heterogeneous nature of the non-IO population, limits the generalizability of our findings as well as the scope of the study. Second, significant differences existed in a number of the baseline characteristics between the IO and non-IO therapy populations, including a higher proportion of patients receiving sorafenib as preceding first-line therapy in the non-IO population and a higher proportion of prior regional therapy in the IO population. The impact of these differences on overall outcomes is unclear. Third, the study was blind to the subsequent lines of therapy subjects may have received after the second-line treatment; these subsequent treatments may be a potential confounding variable. Lastly, although not statistically significant, the non-IO population had a higher proportion of hepatitis C and alcoholic liver disease as underlying etiologies. The impact of this finding remains unclear. 

## 5. Conclusions

To our knowledge, this retrospective comparative study of second-line regimens for HCC is one of the first and largest studies reported to date. No significant difference in survival was identified for patients receiving IO versus non-IO second-line therapy. However, the late survival curve separation favoring IO suggests a delayed IO effect in a subgroup of patients. Many HCC patients will benefit from increased treatment options in the optimal sequence. A great room for improvement remains for identifying an optimal treatment sequence based on disease or tumor molecular profile. Lastly, the recent promising results of the combination of VEGF-targeted therapy plus IO therapy have paved the way for testing of novel anti-VEGF and IO combination therapies in HCC.

## Figures and Tables

**Table 1:** First and subsequent second-line therapies.

1st Line	# (%)	2nd Line	# (%)
**Sorafenib/Lenvatinib**	88 (89.8)	Nivolumab	33 (37.5)
		Pembrolizumab	8 (9.1)
		Regorafenib	18 (20.5)
		Cabozantinib	20 (22.7)
		Ramucirumab	9 (10.2)
**Nivolumab**	10 (10.2)	Sorafenib (100%)	10 (100)

**Table 2:** Baseline Characteristics (comparing patients who received second-line non-IO versus IO).

Baseline Characteristics	Total	Non-IO	IO	*p*-Value
Number	98	57	41	-
Age (mean)	61.9	61.6	62.3	0.76
Gender (Female)	21.4%	17.5%	26.8%	0.26
ECOG * (0–1)	81.6%	80.7%	82.9%	0.45
Child Pugh > B7	16.3%	17.5%	14.6%	0.15
MELD ** score (mean)	14.6	14.7	14.4	0.86
AFP *** (mean)	7982	10,951	3898	0.29
Etiology of Liver Disease				
Hepatitis C	56.1%	57.9%	53.7%	0.67
Hepatitis B	6.1%	5.3%	7.3%	
Alcohol	28.6%	31.6%	22.0%	
Other	9.2%	3.5%	17.1%	
Previous Treatments				
Sorafenib	87.8%	98.2%	73.2%	<0.01
Surgery	5.1%	3.5%	7.3%	0.39
Regional	57.1%	47.4%	70.7%	0.03
Extrahepatic Metastasis	22.4%	17.5%	29.3%	0.73

* Eastern Cooperative Oncology Group (ECOG) performance status scores range from 0 to 5, with higher scores indicating greater disability. ** Model for End-Stage Liver Disease (MELD) is a predictor of survival for patients with advanced liver disease, with higher scores indicating higher mortality. *** Alpha-fetoprotein (AFP).

**Table 3:** Baseline characteristics of the HCV-positive HCC patients (comparing patients who received second-line non-IO versus IO).

Baseline Characteristics	Total	Non-IO	IO	*p*-Value
Number	55	33	22	-
Age (mean)	62.1	61.8	62.4	0.45
Gender (Female)	21.8%	18.2%	22.7%	0.41
ECOG * (0–1)	67.3%	60.6%	77.3%	0.32
Child Pugh > B7	34.5%	33.3%	36.4%	0.42
MELD ** score (mean)	15	15.7	14.3	0.41
AFP *** (mean)	10,024	12,482	7565	0.50
Previous Treatments				
Sorafenib	91%	93.9%	86.4%	0.12
Surgery	3.6%	3.0%	4.5%	0.48
Regional	63.6%	57.6%	72.7%	0.09
Extrahepatic Metastasis	41.8%	39.4%	45.5%	0.44

* Eastern Cooperative Oncology Group (ECOG) performance status scores range from 0 to 5, with higher scores indicating greater disability. ** Model for End-Stage Liver Disease (MELD) is a predictor of survival for patients with advanced liver disease with higher scores indicating higher mortality. *** Alpha-fetoprotein (AFP).
